# Efficient and Secure Encapsulation of a Natural Phase Change Material in Nanofibers Using Coaxial Electrospinning for Sustainable Thermal Energy Storage

**DOI:** 10.1021/acssuschemeng.3c02094

**Published:** 2023-07-27

**Authors:** Dev Patel, Wanying Wei, Harmann Singh, Kai Xu, Christopher Beck, Michael Wildy, John Schossig, Xiao Hu, Dong Choon Hyun, Wenshuai Chen, Ping Lu

**Affiliations:** † Department of Chemistry and Biochemistry, Rowan University, Glassboro, New Jersey 08028, United States; ‡ Department of Physics and Astronomy, Rowan University, Glassboro, New Jersey 08028, United States; § Department of Polymer Science and Engineering, Kyungpook National University, Daegu 41566, South Korea; ∥ Key Laboratory of Bio-based Material Science and Technology, Ministry of Education, Northeast Forestry University, Harbin 150040, China

**Keywords:** lauric acid, polystyrene nanofibers, coaxial electrospinning, phase change materials, thermal energy storage

## Abstract

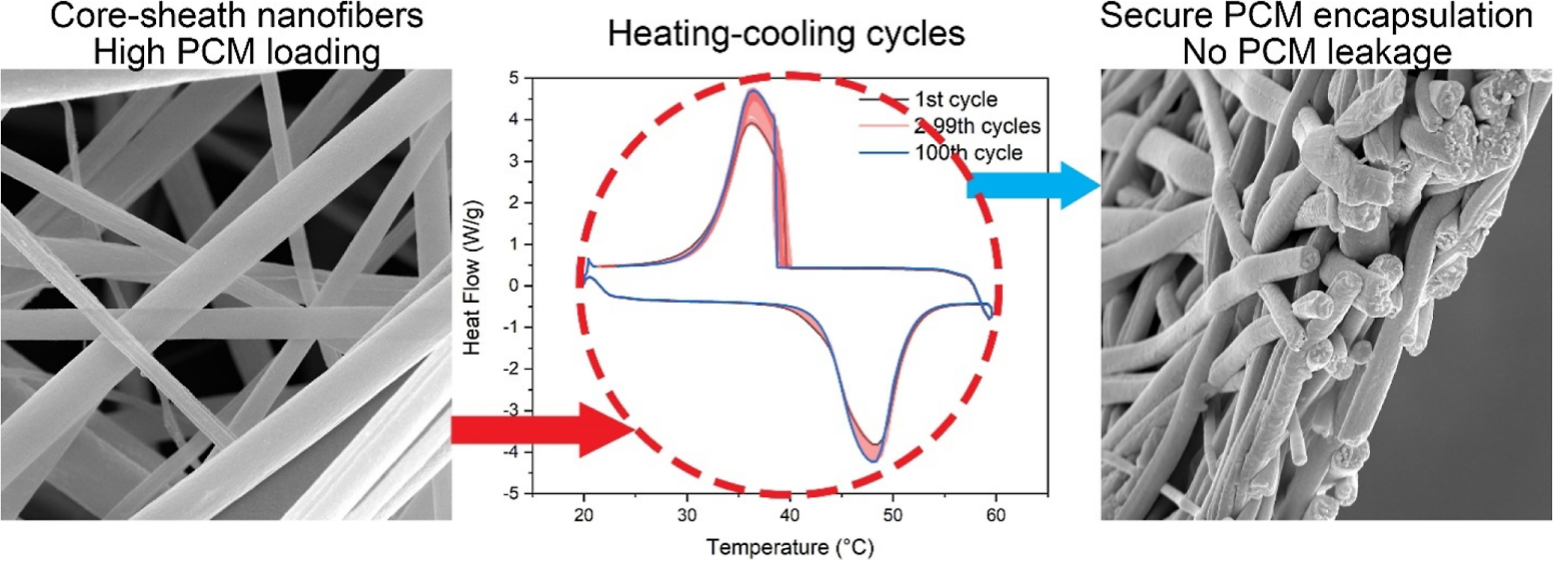

In this study, we present an ecofriendly technique for encapsulating lauric acid (LA), a natural phase change material, within polystyrene (PS) nanofibers through coaxial electrospinning. The resulting LAPS core–sheath nanofibers exhibited a melting enthalpy of up to 136.6 J/g, representing 75.8% of the heat storage capacity of pristine LA (180.2 J/g), a value surpassing all previously reported core–sheath fibers. Scanning electron microscopy revealed uniform LAPS nanofibers free of surface LA until the core LA feed rate reached 1.3 mL/h. As the core LA feed rate increased, the fiber diameter shrank from 2.24 ± 0.31 to 0.58 ± 0.45 μm. Infrared spectra demonstrated a proportional increase in the LA content with rising core LA injection rates. Thermogravimetric analysis found the maximum core LA content in core–sheath nanofibers to be 75.0%. Differential scanning calorimetry thermograms displayed a trend line shift upon LA leakage for LA_1.3_PS nanofibers. LAPS fibers containing 75.0% LA effectively maintained consistent cycling stability and reusability across 100 heating–cooling cycles (20–60 °C) without heat storage deterioration. The core LA remained securely within the PS sheath after 100 cycles, and the LAPS nanofibers retained an excellent structural integrity without rupture. The energy-dense and form-stable LAPS core–sheath nanofibers have great potential for various thermal energy storage applications, such as building insulation, smart textiles, and electronic cooling systems, providing efficient temperature regulation and energy conservation.

## Abstract 2

Coaxial electrospinning was used to efficiently and securely encapsulate a natural phase change material in polymer core–sheath nanofibers for sustainable thermal energy storage.

## Introduction

Natural and biocompatible phase change materials (PCMs) are essential candidates for renewable thermal energy storage, which can help mitigate the global energy crisis associated with climate change and sustainability regulations.^1–4^ PCMs can absorb and release a large amount of latent heat via simple solid-to-liquid and liquid-to-solid physical phase transitions, respectively.^5^ In recent years, organic PCMs, including long-chain alkanes (paraffin), fatty acids, fatty alcohols, and polymers, have attracted significant interest because of their high latent heat of fusion, good thermochemical stability, noncorrosivity, and nontoxicity.^6–8^ Organic PCMs are generally considered a better choice than the corrosive and unstable inorganic PCMs.^9^ Organic PCMs have been used in solar energy storage, industrial waste heat recovery, energy-efficient buildings, temperature-controlled packaging materials, thermo-regulated textiles, Li-ion battery thermal management, etc.^10–12^ Among various organic PCMs, paraffin is the most widely used in industries because of its low cost and high latent heat.^13^ However, paraffin is derived from petroleum and coal, which are nonrenewable and unstainable.^14^ Fatty acids are a viable alternative to paraffin because they are derived from renewable resources such as vegetable oils and animal fats.^15^ Like paraffin, fatty acids are inexpensive and have a high heat-storing capacity.^16^ Further, fatty acids are biodegradable and earth-friendly.^17^ Directly using PCMs for thermal energy storage is disadvantageous because they lose shape stability and leak during melting.^18^ To solve this problem, PCMs are usually encapsulated in suitable shells to form form-stable structures for storing heat.^19^ Additionally, organic PCMs have poor thermal conductivities, which requires PCM composites to have a large surface area and a highly porous structure to increase the heat exchange area and rate between the heat transfer fluid (e.g., air) and PCMs.^20^

Recently, electrospinning has attracted tremendous interest in thermal energy storage because of its proven ability to encapsulate a variety of PCMs into nanofibers.^21^ Further, the nanofiber PCMs achieved much higher PCM loadings than other form-stable PCMs, such as nanocapsules and microcapsules.^22^ Thanks to their large surface area and high porosity, the nanofiber PCMs demonstrated better thermal energy charging and discharging performance than the traditional bulky form-stable PCMs.^23–26^ PCMs can be incorporated into nanofibers by electrospinning blend solutions containing polymers and PCMs.^27^ Our group encapsulated lauric acid (LA) into polystyrene (PS) nanofibers using the blend electrospinning method and achieved high PCM loadings of up to 80%.^24^ However, the blending electrospinning method is not able to control the distribution of PCMs in nanofibers.^28^ As a result, the PCMs located close to or on the nanofiber surface can leak from polymer nanofibers during melting, leading to declined thermal energy storage performance and potential environmental pollution.^29^ To address this problem, coaxial electrospinning has been developed to encapsulate PCMs inside nanofibers by separately feeding a solution containing PCMs and a second solution containing polymers through the inner and outer coaxial needles, respectively.^30,31^ Xia et al. first demonstrated this method using octadecane as the PCM and titanium dioxide–poly(vinylpyrrolidone) (TiO_2_–PVP) as the polymer composite.^32^ Through optimization of the feeding rates of octadecane and TiO_2_–PVP, core–sheath nanofibers consisting of an octadecane core and a TiO_2_–PVP sheath were produced. The maximum octadecane loading in nanofibers reached 45%. Since then, many research groups have used coaxial electrospinning for encapsulating PCMs into nanofibers.^6^ However, the loadings of PCMs in nanofibers have remained relatively low, usually less than or close to 50%.^33^ The low loadings of PCMs using coaxial electrospinning are caused by the significant difference in core and sheath solutions’ spinnability and low compatibility.^34^

To overcome the limitations associated with the low PCM loadings in core–sheath nanofibers and enhance their compatibility, we employed a strategy involving the use of compatible solvents for both core and sheath solutions in the coaxial electrospinning process. Specifically, we selected *N*,*N*-dimethylformamide (DMF) as the solvent for both LA and PS. This choice aimed to minimize the friction between the core and sheath fluids, improving their compatibility and facilitating the formation of stable core–sheath structures during the electrospinning process.^35^ By carefully optimizing the core and sheath feeding rates, we successfully produced LAPS core–sheath nanofibers with significantly improved PCM loadings of up to 75%. This achievement represents a substantial increase in PCM loading compared to previous studies, where the reported values were generally less than or close to 50%.^36^ The use of compatible solvents proved to be a practical approach to addressing the challenges associated with the low loadings of PCMs in core–sheath nanofibers, primarily caused by the significant differences in spinnability and compatibility between core and sheath solutions.^37^ The resulting LAPS core–sheath nanofibers demonstrated excellent thermal energy storage performance with a melting enthalpy of up to 136.6 J/g. Furthermore, and they maintained their structural stability throughout 100 heating–cooling cycles without heat storage deterioration. In addition, the core LA remained securely within the PS sheath after 100 cycles, and the LAPS nanofibers retained excellent structural integrity without rupture. Our approach has great potential for improving PCM-encapsulated nanofibers’ thermal energy storage performance, opening new avenues for their application in various fields.

## Experimental Section

### Chemicals and Materials

A natural fatty acid with a 12-carbon backbone, LA (≥98%), was purchased from Sigma-Aldrich and used as the PCM. Thermochemically durable PS with a molecular weight (*M*_w_) of approximately 350 000 and a number-average molecular weight (*M*_n_) of approximately 170 000 was obtained from Sigma-Aldrich and used as a polymer sheath to encapsulate LA. Common solvents, including anhydrous DMF(≥99.9%), and ethanol (200 proof, ACS grade), were purchased from VWR and employed to dissolve LA and PS. All chemicals were used as received without further purification.

### Encapsulation of LA into PS via Coaxial Electrospinning

**Figure 1:**
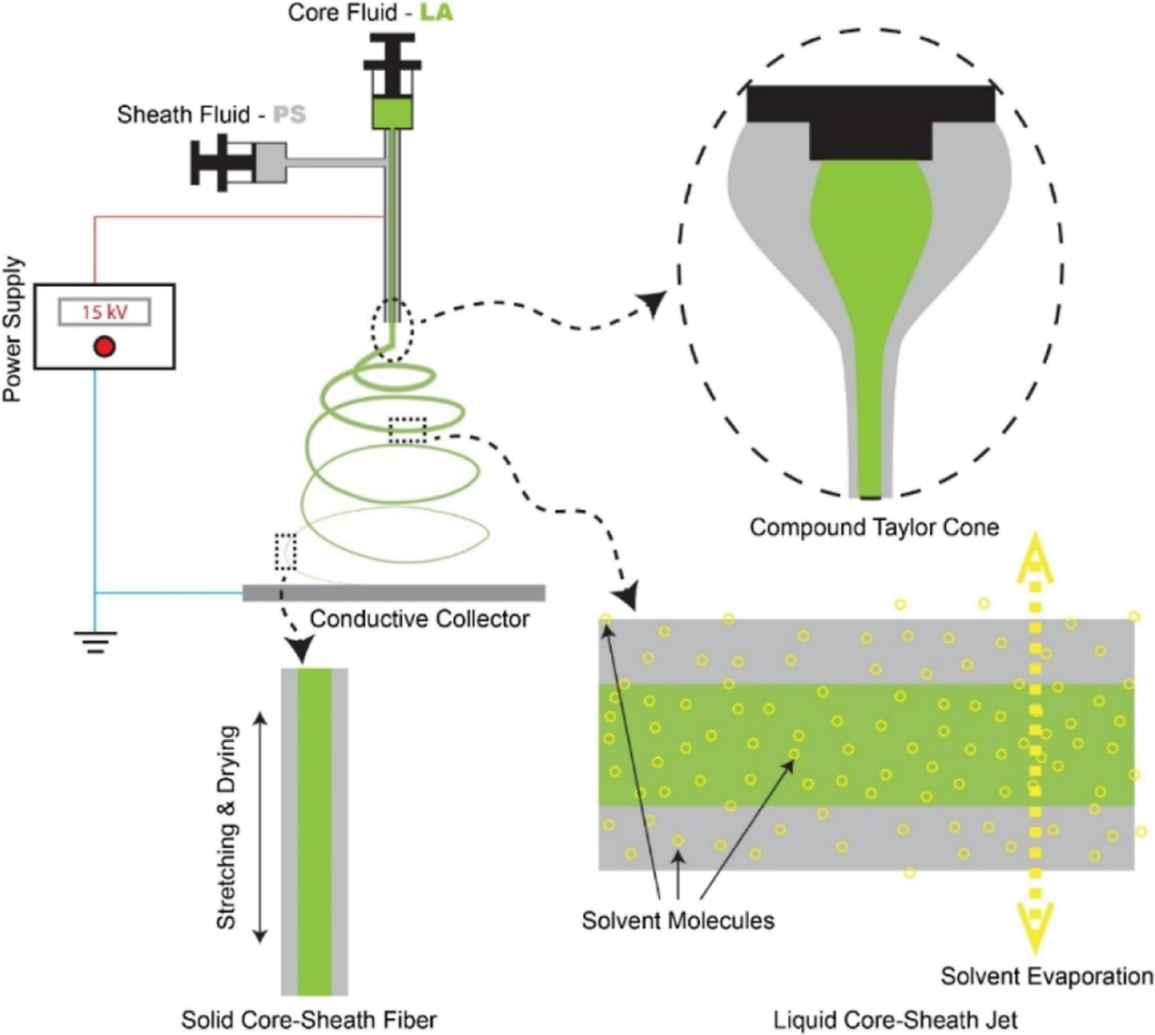
Schematic illustrations showing the coaxial electrospinning process for encapsulating LA into PS by forming LAPS core–sheath nanofibers.

The natural PCM, LA, was encapsulated into PS nanofibers through a controlled coaxial electrospinning process (Figure 1). In a typical experiment, an anhydrous DMF solution containing 20% PS was fed at 1 mL/h into the outer needle of a metal coaxial spinneret, while simultaneously a second anhydrous DMF solution containing LA with a concentration of 0.8 g/mL was fed into the inner needle. The feed rates of the sheath PS solution and the core LA solution were independently controlled by two programmable syringe pumps (Legato 110, KD Scientific). A high-voltage DC power supply (ES30P-5W, Gamma High Voltage Research) was connected to the stainless-steel coaxial spinneret. After charging the spinneret with 15 kV, a liquid jet consisting of core LA solution and sheath PS solution was ejected and spun into solid core–sheath nanofibers due to the bending instability of the charged jet in the electric field and the rapid evaporation of the solvent. After 3 h of electrospinning, a nanofibrous mat (∼2 mm in thickness, 25 cm × 25 cm in length × width) was collected on heavy-duty aluminum foil and positioned 25 cm below the tip of the coaxial spinneret. The obtained core–sheath nanofibers are designated as LA_*x*_PS, where *x* is the feed rate of core LA solution (i.e., 0, 0.1, 0.3, 0.5, 0.7, 1.0, 1.3, and 1.5 mL/h). All electrospinning experiments were conducted at a temperature of 20 ± 2 °C and a relative humidity of 50 ± 3%. The temperature was controlled by the laboratory’s central air conditioning system, and the humidity was controlled using an industrial-grade humidifier/dehumidifier. The obtained nanofibers were dried in a vacuum oven for 24 h at room temperature before subsequent experiments and characterizations.

### Thermal and Structural Stability

The thermal stability of form-stable nanofiber PCM was evaluated by two methods: (1) The nanofiber mats were continuously heated at 60 °C for a week using a precision oven (VWR). (2) The nanofiber mats were repeatedly heated and cooled in the temperature range from 20 to 60 °C for 100 cycles. The underneath Whatman filter papers used to support nanofiber mats in these experiments were visually inspected to determine the contact leakage of LA (melting point: 46 °C) after its phase transition from solid to liquid and vice versa. Using ethanol, nanofiber PCM’s structural stability and polymer sheath integrity were determined after selectively removing LA from the core–sheath nanofibers. Ethanol is a good solvent for LA and a nonsolvent for PS. A piece of nanofiber mat (4 cm × 4 cm × 2 mm) was immersed in 10 mL of ethanol for 20 min with continuous agitation (300 rpm) using an orbital shaker (LSE, Corning). Next, ethanol and dissolved LA were removed by vacuum filtration using a Büchner funnel. The above ethanol extraction and filtration were repeated two more times to remove LA completely from the nanofibers completely. The heated and washed nanofibers were dried in a vacuum oven for 24 h at room temperature before subsequent characterizations.

### Characterization

The surface morphology and internal structure of the as-spun, heated, and washed nanofibers were imaged with a high-resolution field-emission scanning electron microscope (Apreo, FEI). Nanofibers were fractured in liquid nitrogen (−195.8 °C) to reveal the internal structure and vacuum-dried. All samples were sputter-coated with gold (30–120 s, depending on the sample) to enhance their electric conductivity. Representative images of samples were taken at a 6 mm analytical working distance using a 10 kV accelerating voltage and 0.40 nA beam current. Nanofiber size was measured with imageJ (NIH) using SEM images, and fiber size distribution was statistically analyzed by OriginPro (OriginLab). The chemical composition of nanofibers was analyzed by using a PerkinElmer Frontier infrared spectrometer with the attenuated total reflection (ATR) technique. The absorbance spectra of nanofibers were recorded in 4000–650 cm^–1^ at a 4 cm^–1^ resolution, averaging 128 scans. A simultaneous thermal analyzer determined the precise LA weight percentage in the LAPS core–sheath nanofibers (DSC-TGA, TA SDT 600). In a typical measurement, around 10 mg of nanofibers was heated in an alumina pan from room temperature (∼20 °C) to 600 °C at a 10 °C/min ramp rate in dry nitrogen (purging rate: 100 mL/min). The recorded thermogram was used to calculate the LA content based on the weight loss during thermal degradation. The thermal energy storage capacity of nanofiber PCMs was measured by a differential scanning calorimeter (TA Q100) coupled with a refrigerated cooling system (−90–550 °C, TA RCS90). In a standard measurement, 5–10 mg of nanofibers, sealed in a crimpled aluminum pan, underwent heating–cooling cycles in 20–70 °C at a 10 °C/min ramp rate under 100 mL/min dry nitrogen purging. The obtained thermograms were used to derive the enthalpies of melting/crystallization of pure LA (Δ*H*_LA_) and LAPS nanofibers (Δ*H*_LAPS_). The thermal energy storage capacity of LAPS nanofibers was calculated using the following equation
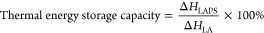


## Results and Discussion

### Encapsulation of LA into PS Nanofibers via the Coaxial Electrospinning Process

The schematic illustration in Figure 1 demonstrates the coaxial electrospinning process used to encapsulate LA into PS by forming LAPS core–sheath nanofibers. The major components of the coaxial electrospinning setup include the coaxial spinneret, high voltage power supply, and conductive collector.^38–40^ The core fluid consisted of 0.8 g/mL LA in DMF, while the sheath fluid comprised 20% PS in DMF. At the tip of the needles, a compound Taylor cone formed when charged with a high voltage, enabling the core–sheath structure. As the process continued, diffusion and evaporation of solvent DMF occurred within the liquid core–sheath jet. Finally, solid fibers were formed after stretching and drying on the fly in an electric field, resulting in the LAPS core–sheath nanofibers with different LA contents.

**Figure 2:**
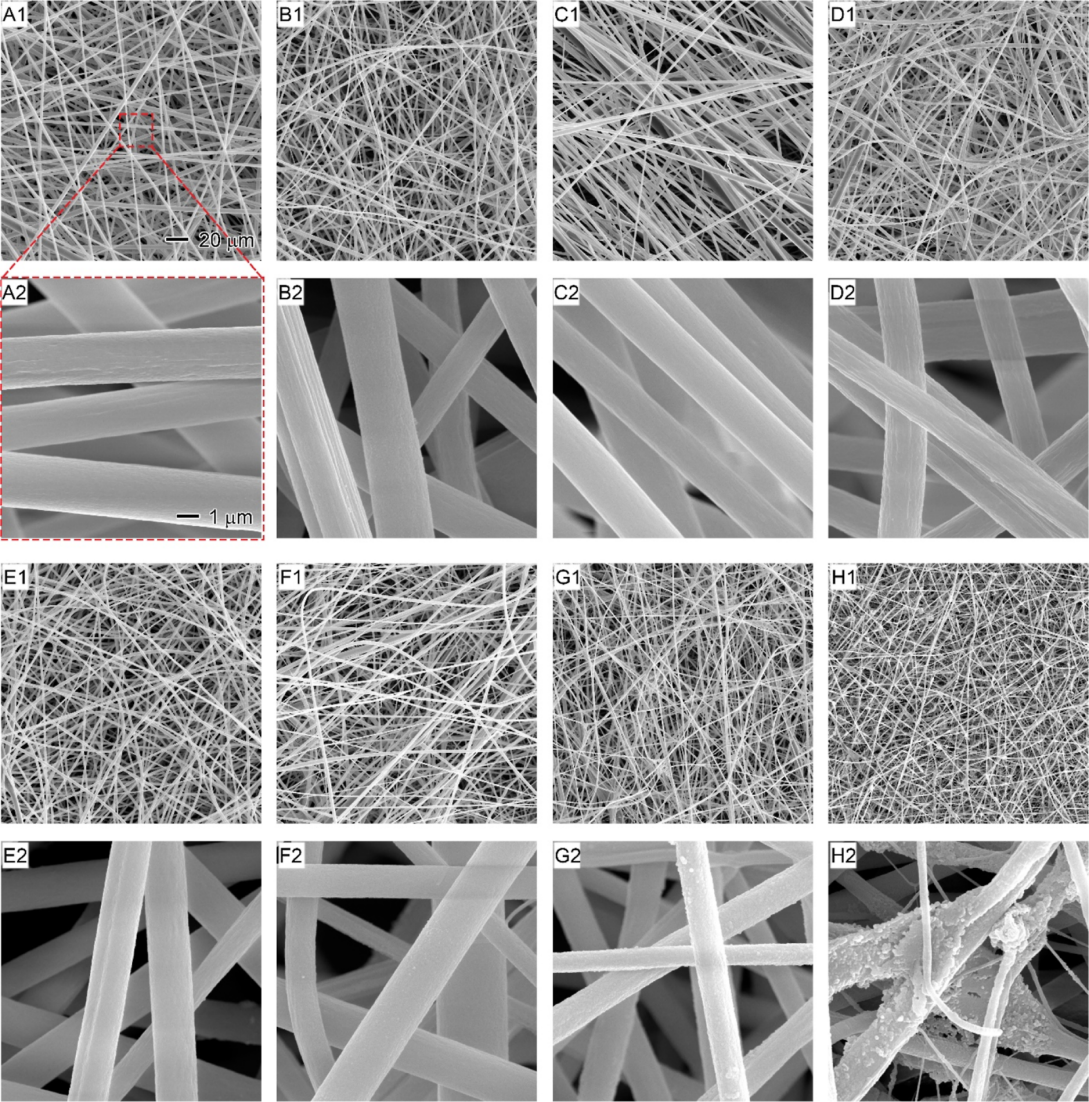
SEM images showing (1) the overview of fiber quality and (2) the individual fibers using different core LA feed rates: (A) 0 0.1, (C) 0.3, (D) 0.5, (E) 0.7, (F) 1.0, (G) 1.3, and (H) 1.5 mL/h. The sheath PS feed rate is 1.0 mL/h. The 20 μm scale bar in (A1) applies to all images with the label “1” and the 1 μm scale bar in (A2) applies to all images with the label “2”.

By controlling the feed rates and optimizing the coaxial electrospinning process, smooth and uniform LAPS core–sheath nanofibers with different LA contents were obtained. The SEM images in Figure 2 show the morphology of the produced LAPS nanofibers by using different core LA feed rates ranging from 0 to 1.5 mL/h while maintaining a constant sheath PS feed rate of 1.0 mL/h. The low-magnification images (labeled “1”: A1–H1) provide insights into the overall nanofiber quality, while the high-magnification images (labeled “2”: A2–H2) offer a closer look at individual nanofibers. The core LA injection rate played a critical role in maintaining the quality and morphology of the LAPS core–sheath nanofibers. The nanofibers exhibited a cylindrical shape and smooth surface when core LA injection rates were kept within 0 to 1.0 mL/h (Figure 2A–F). This observation suggests that the coaxial electrospinning process was stable, potentially due to using the same solvent, DMF, for both core and sheath solutions. The uniformity and compatibility of the solvent likely minimized the interfacial friction between the core and sheath fluids, resulting in high spinnability and enhanced fiber quality. However, as the core LA feed rate increased to 1.3 mL/h, tiny solid LA particles were observed on the surface of the nanofibers (Figure 2G). This indicates that the electrospinning process might be reaching its limit for the successful encapsulation of LA. At an even higher rate of 1.5 mL/h, larger LA aggregates formed and coated the fibers (Figure 2H), suggesting that excessive LA feed rates could lead to saturation of the sheath PS solution and compromise the structural integrity of the resulting nanofibers.

**Figure 3:**
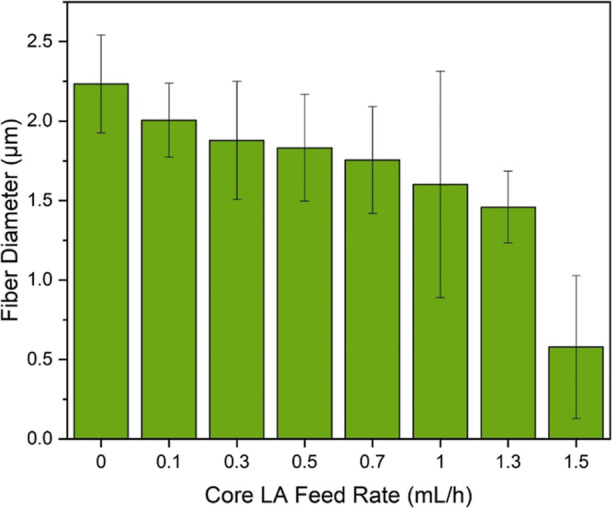
Effect of the core LA feed rate on the diameters of LAPS core–sheath fibers.

Figure 3 demonstrates the impact of varying core LA feed rates on the diameters of the LAPS core–sheath nanofibers. As the core LA feed rate increased from 0 to 1.5 mL/h, the fiber diameter decreased from 2.24 to 0.58 μm. This trend can be attributed to a higher core feed rate, resulting in a thinner sheath layer due to increased encapsulated LA. This thinner sheath layer, in turn, leads to a decrease in the overall diameter of the LAPS core–sheath fibers. Moreover, it is worth noting that the core fluid, LA, might also serve as a plasticizer in the electrospinning process. This could contribute to the reduction in fiber diameter as the presence of a plasticizer tends to lower the viscosity and intermolecular friction within the polymer solution, subsequently leading to finer fibers. However, a significant decrease in fiber diameter was observed when the core LA feed rate increased from 1.3 to 1.5 mL/h, dropping from 1.46 to 0.58 μm. This abrupt change suggests a failed encapsulation of LA inside the PS sheath at the highest core LA feed rate. The failure in encapsulation could be due to the higher core feed rate exceeding the capacity of the sheath to effectively encapsulate the LA, causing the LA to leak or aggregate on the fiber surface instead of being fully encapsulated within the PS sheath (Figure 2H).

**Figure 4:**
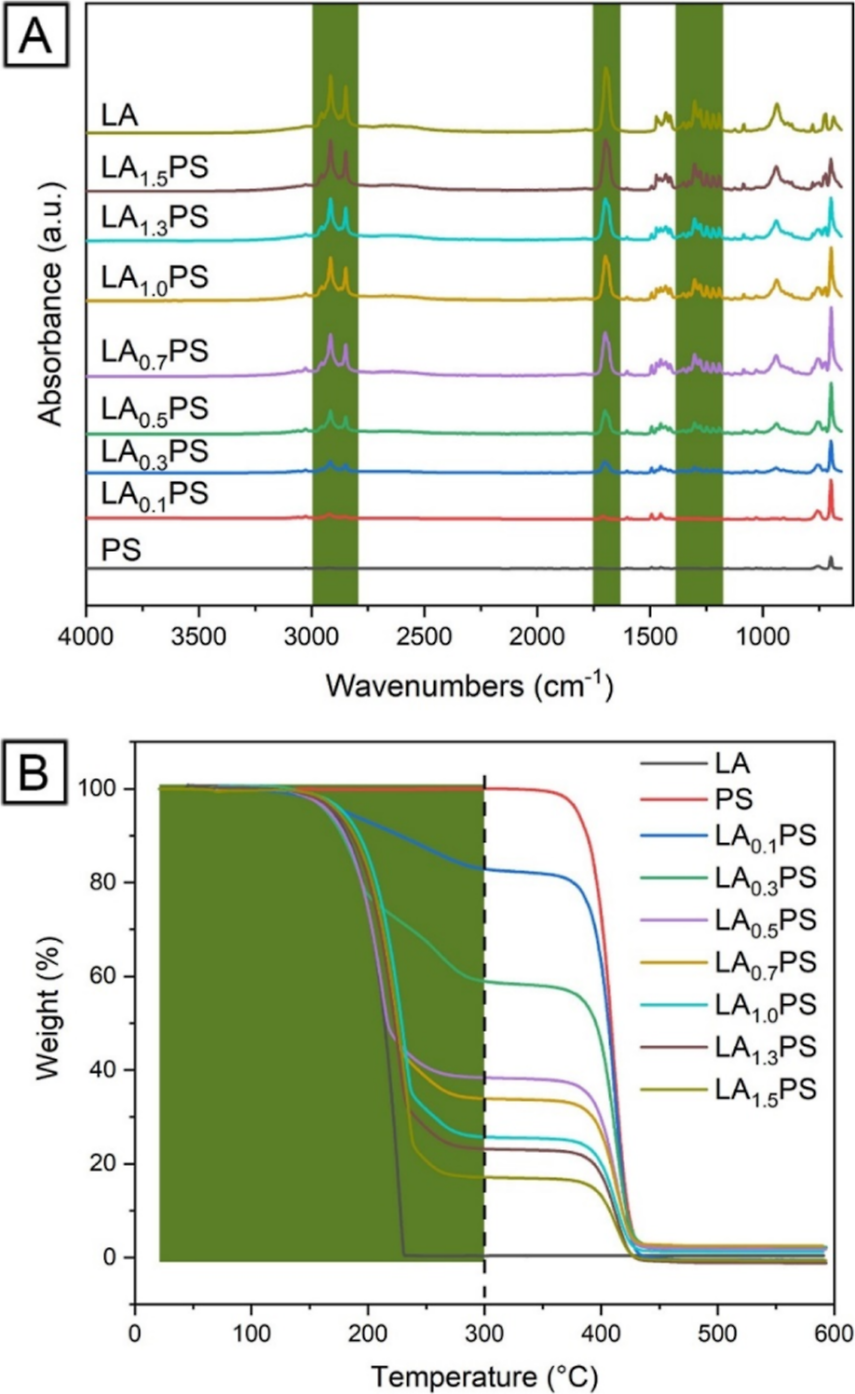
Infrared spectra (A) and TGA thermograms (B) of PS, LA, and LAPS core–sheath fibers prepared by using different core LA feed rates.

The presence of LA in the LAPS core–sheath fibers was further confirmed through ATR–FTIR analysis, as shown in Figure 4A. The distinct characteristic peaks of LA were observed at 2915 cm^–1^ (–CH_3_), 2848 cm^–1^ (–CH_2_–), 1695 cm^–1^ (C=O), 1470–1410 cm^–1^ (–CH_3_ and –CH_2_–), and 1085 cm^–1^ (C–O).^41^ On the other hand, PS exhibited bands at 3025 cm^–1^ (aromatic CH), 2920 cm^–1^ (aliphatic CH_2_ and CH), and 1492 and 1451 cm^–1^ (aromatic CC).^42^ Among these absorbance peaks, the carbonyl peak at 1695 cm^–1^ serves as a unique identifier for estimating the quantity of LA present in the composite fibers.^24–26^Figure 4A shows that the carbonyl peak intensity increased with the rise in core LA injection rates, ranging from 0 to 1.5 mL/h. This observation is further supported by comparing the absorbance peaks of the two reference samples, pure PS and LA. As the core injection rate increases, the increasing intensity of the carbonyl peak indicates a higher concentration of LA encapsulated within the composite fibers. This finding verifies the successful incorporation of LA into the LAPS core–sheath fibers and demonstrates the capacity to adjust and control the LA content by altering the core injection rate during the coaxial electrospinning process.

The LA content in the LAPS core–sheath fibers was further determined through TGA analysis, as depicted in Figure 4B. The decomposition of LA occurred in a single step, exhibiting a weight loss of 98.0% in the temperature range 25–300 °C. Similarly, PS demonstrated a one-step decomposition process, with nearly 100% weight loss between 300 and 600 °C. Notably, there was no overlap between PS and LA degradation thermograms, indicating distinct thermal degradation behaviors for each component. A two-step decomposition process was observed in the case of LAPS core–sheath fibers. The first step, occurring in the 25–300 °C range, was attributed to the degradation of the encapsulated LA. In contrast, the second step, taking place between 300 and 600 °C, was associated with the degradation of the PS sheath. Significantly, the encapsulation of LA within the PS hollow fibers did not adversely affect their respective thermal properties. Upon closer examination, it was observed that the weight loss corresponding to the first step, due to the thermal degradation of LA, increased as the core LA injection rates were raised from 0.1 to 1.5 mL/h. This finding suggests that the LA content within the LAPS core–sheath fibers can be effectively controlled by adjusting the core injection rate during the coaxial electrospinning process, thereby enabling the fine-tuning of the fibers’ thermal properties for specific applications.

Table 1 presents the LA contents in the LAPS core–sheath fibers as determined by theoretical calculations and experimental measurements. The theoretical LA content was derived from the core and sheath fluid feed rates, while the measured LA content was obtained from the weight loss in the 25–300 °C range during the TGA. Upon analyzing the data, it is apparent that the measured LA content generally follows the trend of the theoretical LA content, increasing as the core LA feed rate increases. However, discrepancies between the theoretical and measured LA contents were observed, particularly at lower core LA feed rates. For instance, at a core LA feed rate of 0.1 mL/h, the theoretical LA content is 25.3%, whereas the measured LA content is 18.2 ± 1.9%. This discrepancy could be attributed to experimental factors, such as the nonequilibrium flow of LA during the electrospinning process. As the core LA feed rate increases, the differences between the theoretical and measured LA contents become smaller, indicating a more effective encapsulation of LA in the core–sheath fibers. At higher core LA feed rates, such as 1.0 mL/h, the measured LA content (75.0 ± 5.0%) is closer to the theoretical values (77.2%), suggesting a more consistent and efficient encapsulation process at these feed rates.

**Table 1:** LA Contents in LAPS Core–Sheath Fibers from Theoretical Calculations and Measurements

core LA feed rate (mL/h)	theoretical LA content (%)	measured LA content (%)
0	0	0
0.1	25.3	18.2 ± 1.9
0.3	50.4	42.6 ± 3.6
0.5	62.9	61.9 ± 5.9
0.7	70.4	66.2 ± 6.2
1.0	77.2	75.0 ± 5.0
1.3	81.5	77.3 ± 6.4
1.5	83.6	82.8 ± 3.8

**Figure 5:**
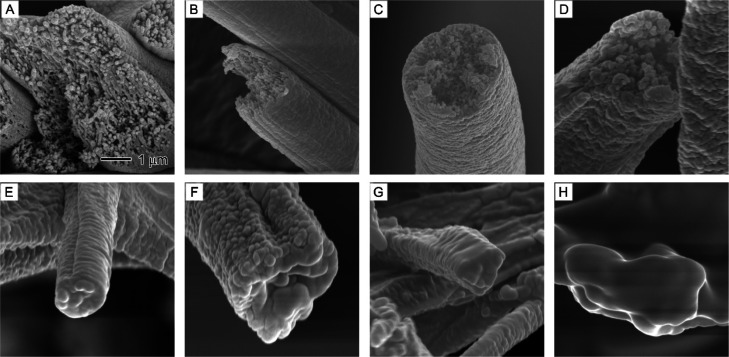
SEM images showing the cross sections of LAPS core–sheath fibers with different LA contents: (A) 0, (B) 18.2 ± 1.9, (C) 42.6 ± 3.6, (D) 61.9 ± 5.9, (E) 66.2 ± 6.2, (F) 75.0 + 5.0, (G) 77.3 + 6.4, and (H) 82.8 ± 3.8%. The 1 μm scale bar in (A) applies to all images.

Figure 5 provides insights into the distribution of LA in LAPS composite fibers by examining their surface and cross sections with varying LA contents. The SEM images show that the core LA injection rate influenced the distribution and encapsulation of LA within the fibers. A highly porous interior was observed for the PS fibers without LA (Figure 5A). In the case of LAPS core–sheath fibers obtained with 0.1, 0.3, and 0.5 g/mL core LA injection rates (Figure 5B–D), the cross sections still exhibit porous structures, indicating that the interiors of these fibers were only partially filled with LA. This partial filling is consistent with the lower LA contents for these injection rates, as presented in Table 1. When the core LA feed rate was increased to 0.7 g/mL (Figure 5E), the fiber interior became solid and nonporous, suggesting a more effective encapsulation of LA. This observation aligns with the increase in measured LA content to 66.2 ± 6.2% at this injection rate. As the core feed rate was further increased to 1.0 mL/h (Figure 5F), the packing of LA inside LAPS core–sheath fibers became denser, resulting in an even higher measured LA content of 75.0 ± 5.0%. However, when the core feed rate was raised to 1.3 mL/h, LA leakage onto the surface of the composite nanofibers was observed, indicating a less controlled encapsulation process. This leakage is likely responsible for the minor increase in measured LA content to 77.3 ± 6.4% at this injection rate. Eventually, at an even higher feed rate, a giant LA monolith was observed in the membrane, which suggests that the encapsulation process was overwhelmed and was no longer effective.

**Figure 6:**
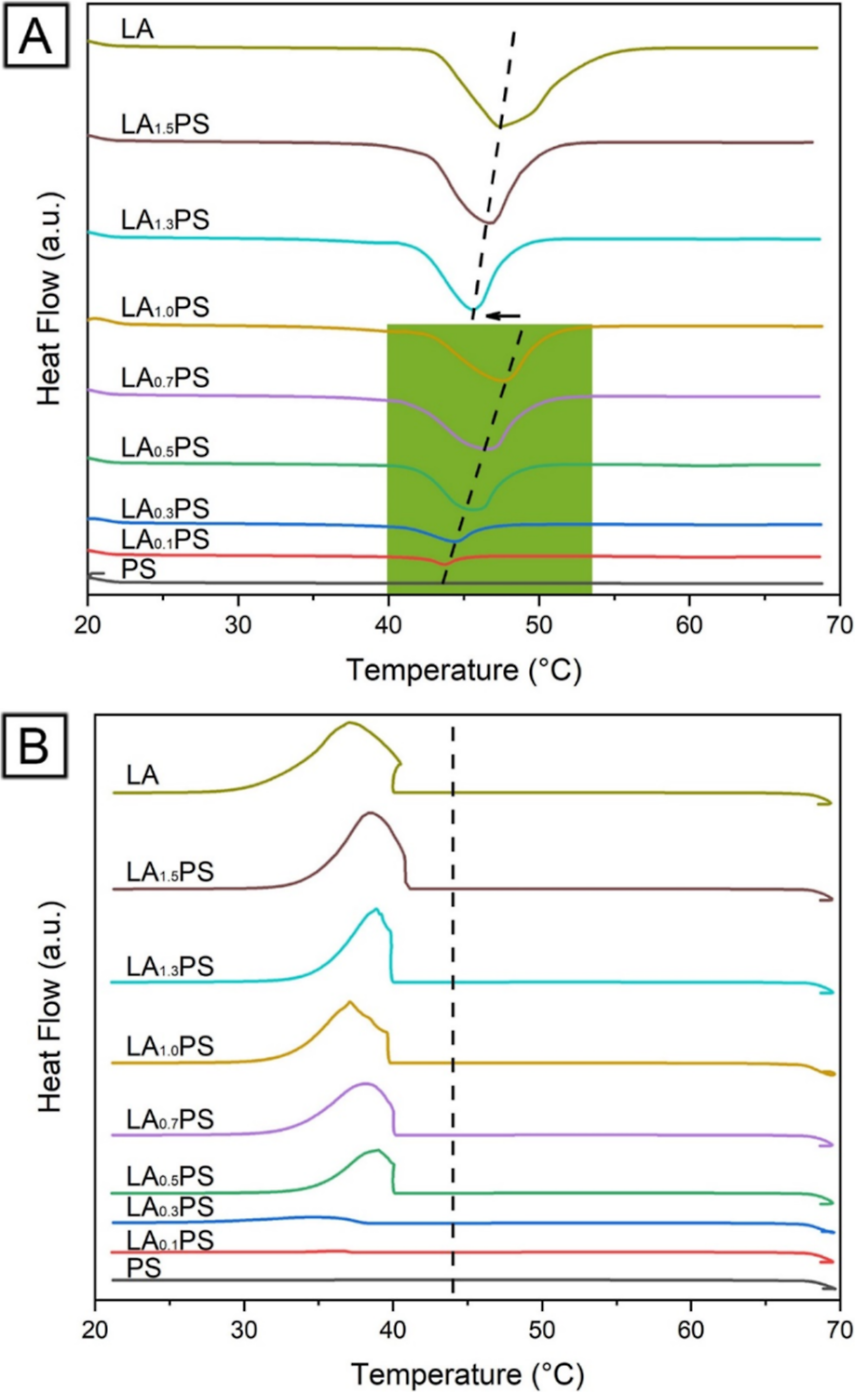
DSC thermograms of pristine LA powder, PS fibers, and LAPS core–sheath fibers during (A) heating from 20 to 70 °C and (B) cooling from 70 to 20 °C.

Figure 6 presents the DSC thermograms of LAPS core–sheath nanofibers with varying LA contents using pure PS and LA as references. Pure PS exhibited no thermal changes between 20 and 70 °C, while pure LA displayed distinct melting and crystallization peaks. During the heating process, a unimodal endothermic peak at 43.69 °C was observed for LA_0.1_PS (Figure 6A), which was attributed to the melting of encapsulated LA (*T*_m_). As the LA content increases, the endothermic peaks of LAPS composite fibers shift toward higher temperatures, reaching 47.60 °C for LA_1.0_PS. This increase in melting peak temperature is associated with the growth in the size of encapsulated LA crystal domains within LAPS core–sheath nanofibers, as larger LA crystals require more heat to melt. An abrupt shift toward a lower temperature (45.61 °C) was noted for LA_1.3_PS, followed by a slight increase to 46.62 °C for LA_1.5_PS. This unexpected shift could be due to the formation of LA domains near or on the nanofiber surface, as observed in the SEM images. Exposed LA domains on the nanofiber surface can melt faster than encapsulated LA because it takes extra time for the heat to travel to the core LA. According to the measurements, the enthalpy of melting (Δ*H*_m_) of pristine LA is 180.2 J/g, and its enthalpy of crystallization (Δ*H*_c_) is 180.0 J/g. Both values can be a reference for determining the thermal energy storage capacity of LAPS composite nanofibers’ thermal energy storage capacity. For simplicity, we focused on the enthalpy of melting (Δ*H*_m_) in our analysis. The melting enthalpy increased with the LA content in LAPS core–sheath nanofibers, starting at 32.9 J/g for LA_0.1_PS and reaching 136.6 J/g for LA_1.0_PS. Further increases for LA_1.3_PS and LA_1.5_PS are related to leaked LA, as shown in Figures 2 and 5.

During the cooling process, LA crystallization began at around 40 °C (*T*_c_), which is lower than its melting temperature (*T*_m_ = 47.4 °C). A crystallization process typically consists of two steps: nuclei formation and crystal growth. When melted LA cools to a temperature where the free enthalpy of the crystal becomes smaller than the free enthalpy of the melt, crystallization occurs if a sufficient number of nuclei are present. However, the melt can be maintained below its freezing (or melting) point without solidifying if there are insufficient nuclei, a phenomenon called the supercooling effect. In this case, the crystallization temperature is lower than the melting temperature (*T*_c_ < *T*_m_), as evidenced in Figure 6B. Supercooling is a characteristic phenomenon commonly observed in organic PCMs.

### Thermal Performance and Structural Stability of LAPS Core–Sheath Nanofibers

**Figure 7:**
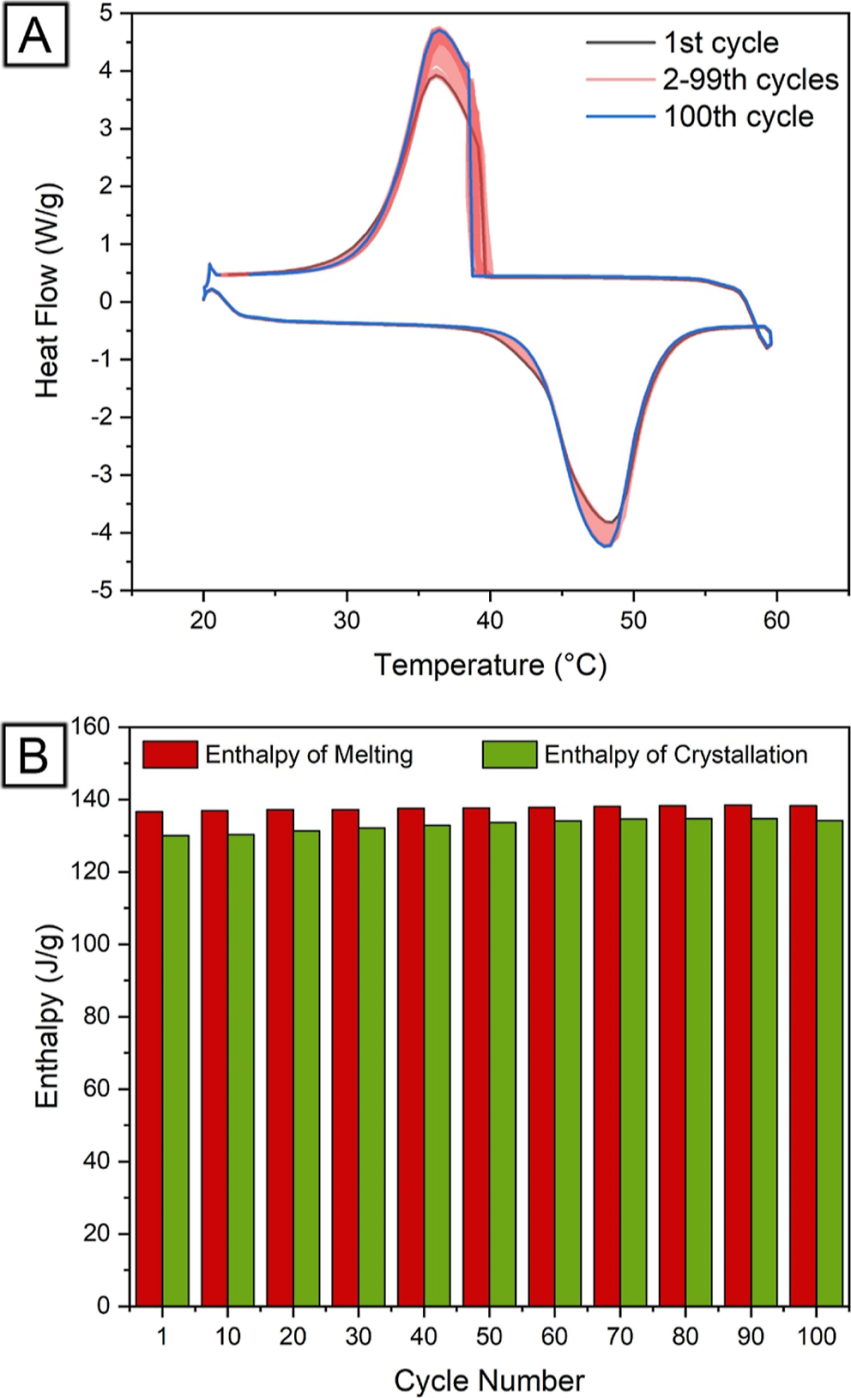
(A) DSC thermograms of LA_1.0_PS core–sheath fibers during 100 continuous heating–cooling cycles. (B) Enthalpy values of melting and crystallization at different cycles.

The DSC thermograms of LA_1.0_PS during 100 continuous heating–cooling cycles (Figure 7A) provide evidence of the material’s cycling stability. The slight shift in the melting peak temperature from 48.49 °C for the first cycle to 47.96 °C for the 100th cycle could be attributed to the initial redistribution of LA within the nanofibers due to melting during the first cycle. This slight change in the melting peak temperature demonstrates that the LA_1.0_PS core–sheath nanofibers maintained their thermal properties throughout multiple heating–cooling cycles. Moreover, the crystallization peaks remained relatively consistent throughout the cycles, with only a minor change from 36.22 °C for the first crystallization peak to 36.43 °C for the 100th crystallization peak. This further supports the notion that the LA_1.0_PS core–sheath nanofibers had excellent cycling stability, maintaining a consistent crystallization behavior even after repeated phase changes.

The cycling stability of LA_1.0_PS core–sheath nanofibers was further analyzed by using the enthalpies of melting and crystallization obtained from 100 continuous heating–cooling cycles (Figure 7B). A slight increase in the enthalpy of melting was observed, ranging from 136.6 J/g in the first cycle to 138.3 J/g in the 100th cycle. Similarly, the crystallization enthalpy increased from 130 to 134.2 J/g over 100 cycles. This increase could be attributed to a more homogeneous distribution of LA within the core–sheath nanofibers during the initial heating–cooling cycles, leading to more efficient energy storage and release in the subsequent cycles. The minimal variation in melting and crystallization enthalpies suggests that the LA_1.0_PS core–sheath nanofibers exhibit excellent cycling stability. Compared with our previous work on LAPS blend fibers, which achieved a heat storage capacity of 78.4%, the LA_1.0_PS core–sheath nanofibers encapsulated with 75.0% (±5.0%) LA demonstrated a slightly lower heat storage capacity of 75.8%. Despite this difference, the core–sheath structure of the LA_1.0_PS nanofibers provided a more secure encapsulation of LA, ensuring better control over its distribution compared to that of the blend fibers.

The crystallization enthalpy is consistently smaller than the melting enthalpy for each cycle. One possible reason for this discrepancy is the energy loss due to thermal expansion during the solid–liquid phase change. As LA transitions from solid to liquid, the material undergoes thermal expansion, which requires energy to overcome intermolecular forces and allow the material to expand. This energy expenditure results in a crystallization enthalpy slightly lower than the melting enthalpy. Despite the difference in enthalpy values, the relatively stable and close enthalpy values of melting and crystallization throughout the cycles indicate the excellent cycling stability of LA_1.0_PS core–sheath nanofibers. In addition, the secure encapsulation of LA within the core–sheath structure ensures better control over its distribution. It minimizes potential issues such as leakage and phase separation, which might affect the thermal energy storage performance of the fibers.

**Figure 8:**
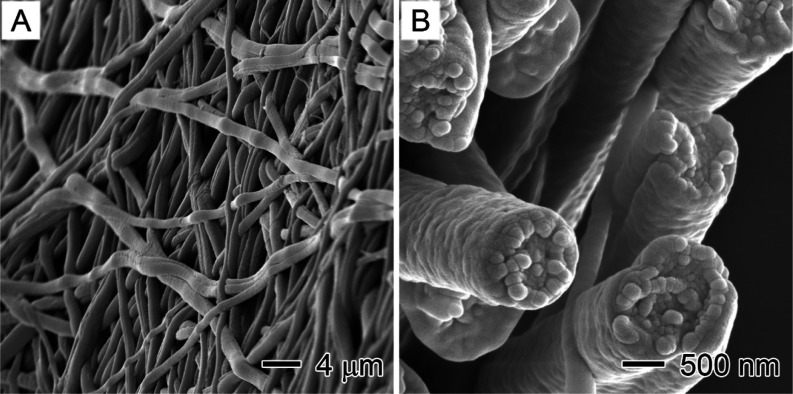
SEM images showing the (A) membrane and (B) cross sections of LA_1.0_PS core–sheath fibers after 100 continuous heating–cooling cycles.

The SEM measurement of the LA_1.0_PS core–sheath nanofibers after 100 continuous heating–cooling cycles at 20–60 °C (Figure 8) demonstrates their excellent retention of the encapsulated PCM, a critical factor for their practical application in thermal energy storage. The polymer matrix softened as the LA melted during the heating process, increasing the nanofibers’ packing density within the membrane. This increased packing density allowed individual nanofibers to support each other, contributing to the stability and structural integrity of the nanofibers. Importantly, no leakage of LA from the nanofibers was observed during SEM examination, as evidenced by the absence of any LA droplets on the nanofiber surface or within the interfiber voids (Figure 8A). This indicates that the LA was securely encapsulated within the core–sheath nanofibers, even after multiple heating–cooling cycles. The LA domains were tightly packed inside the LA_1.0_PS core–sheath nanofibers. The combination of the polymer sheath and the surface tension of liquid LA in the nanocapillary effectively locked the LA inside the nanofibers upon melting. Additionally, no contact leakage of LA was detected from the LA_1.0_PS core–sheath nanofibers after heating the membrane on filter papers continuously at 60 °C for 1 week. This further underscores the reliable encapsulation of LA within the nanofibers and the material’s potential for long-term use in thermal energy storage applications.

**Figure 9:**
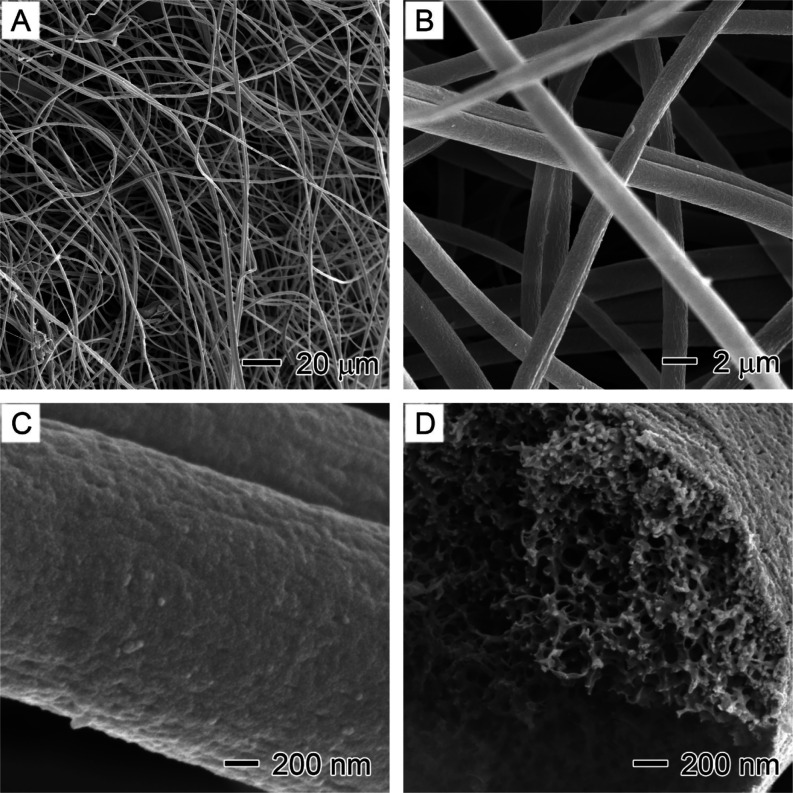
SEM images showing the thermally treated LA_1.0_PS core–sheath fibers after the removal of the inner LA phase using ethanol extraction: (A) overview, (B) interfiber space, (C) fiber surface, and (D) cross section.

Figure 9 shows the thermally treated LA_1.0_PS core–sheath fibers after removing encapsulated LA. The PS matrix maintained its structural stability after repeated heating and cooling cycles, even with volume expansion and contraction during these cycles. After the encapsulated LA was removed from the samples by ethanol extraction, the resulting PS fibers had an average diameter of 1.68 ± 0.24 μm, which is very close to that of the original LA_1.0_PS fibers (1.60 ± 0.71 μm). This suggests that the fibers can be regenerated and reused without significantly changing their dimensions. Once the LA was removed, the packing of fibers returned to a state similar to that of as-spun fibers (Figure 9A,B), with an increased distance between the fibers. This observation and the absence of cavities on the fiber surface (Figure 9C) confirm that the LA was fully encapsulated within the core region of the core–sheath fibers without diffusion into the polymer sheath.

Considering the density changes of PS and LA with temperature, it is evident that the PS matrix can accommodate the volume expansion of LA during heating as the density of PS remains relatively constant while the density of LA decreases. This resulted in a 116% volume expansion of LA within the PS hollow fibers. Moreover, no broken fibers were observed in the samples, demonstrating the PS matrix’s high thermal–mechanical strength and flexibility. Furthermore, the interior pores that were filled with LA nanodomains in the LA_1.0_PS core–sheath fibers remained intact after the harsh thermal treatment (Figure 9D), further highlighting the exceptional structural stability of the PS material for long-term thermal energy storage applications.

## Conclusions

In conclusion, using the coaxial electrospinning technique, our study successfully demonstrated the development and characterization of LAPS core–sheath nanofibers as an effective thermal energy storage material. This green and facile approach allowed for precise control of the encapsulation of LA within PS, ensuring secure retention of the phase change material during repeated heating and cooling cycles. SEM, FTIR, TGA, and DSC results revealed a direct correlation between the core LA injection rate and LA content within the fibers. The LA_1.0_PS core–sheath nanofibers showed the highest thermal energy storage capacity of 136.6 J/g, which is 75.8% of the heat storage capacity of pure LA (180.2 J/g). This performance is close to that of LAPS blend fibers reported in our previous work^24–26^ while offering the added advantage of improved control over LA distribution within the fibers. The core–sheath nanofibers demonstrated excellent cycling stability, as evidenced by the minimal variation in the melting and crystallization peaks during 100 continuous heating–cooling cycles. Furthermore, the structural stability of the PS matrix was confirmed after repeated heating–cooling cycles with no significant changes in fiber dimensions or LA leakage observed. In addition, the PS matrix exhibited high thermal–mechanical strength and flexibility, accommodating LA’s 116% volume expansion during heating without any visible damage or breakage. The interior pores remained intact after a harsh thermal treatment, highlighting the exceptional structural stability of the PS material for long-term thermal energy storage applications. Our findings underscore the potential of LAPS core–sheath nanofibers as a promising thermal energy storage solution with secure encapsulation of the phase change material, high thermal energy storage capacity, and excellent structural stability. Furthermore, the possibility of regeneration and reuse of these fibers makes them an attractive option for sustainable and efficient energy storage applications.
